# Identification of biomarkers that distinguish chemical contaminants based on gene expression profiles

**DOI:** 10.1186/1471-2164-15-248

**Published:** 2014-03-31

**Authors:** Xiaomou Wei, Junmei Ai, Youping Deng, Xin Guan, David R Johnson, Choo Y Ang, Chaoyang Zhang, Edward J Perkins

**Affiliations:** 1 Department of Lab Science, Fourth Hospital Affiliated to Guangxi Medical University, Liuzhou, China; 2 Department of Internal Medicine, Rush University Cancer Center, Rush University Medical Center, Kidston House, 630 S. Hermitage Ave. Room 408, Chicago, IL 60612, USA; 3 SpecPro Inc, Vicksburg, MS 39180, USA; 4 US Army Engineer Research and Development Center, 3909 Halls Ferry Road, Vicksburg, MS 39180, USA; 5 Conestoga-Rovers & Associates, 2290 Springlake Road, Suite 108, Dallas, TX 75234, USA; 6 School of Computing, University of Southern Mississippi, Hattiesburg, MS 39406, USA

**Keywords:** Biomarker, Microarray, Hepatocytes, Chemical, Classification

## Abstract

**Background:**

High throughput transcriptomics profiles such as those generated using microarrays have been useful in identifying biomarkers for different classification and toxicity prediction purposes. Here, we investigated the use of microarrays to predict chemical toxicants and their possible mechanisms of action.

**Results:**

In this study, *in vitro* cultures of primary rat hepatocytes were exposed to 105 chemicals and vehicle controls, representing 14 compound classes. We comprehensively compared various normalization of gene expression profiles, feature selection and classification algorithms for the classification of these 105 chemicals into14 compound classes. We found that normalization had little effect on the averaged classification accuracy. Two support vector machine (SVM) methods, LibSVM and sequential minimal optimization, had better classification performance than other methods. SVM recursive feature selection (SVM-RFE) had the highest overfitting rate when an independent dataset was used for a prediction. Therefore, we developed a new feature selection algorithm called gradient method that had a relatively high training classification as well as prediction accuracy with the lowest overfitting rate of the methods tested. Analysis of biomarkers that distinguished the 14 classes of compounds identified a group of genes principally involved in cell cycle function that were significantly downregulated by metal and inflammatory compounds, but were induced by anti-microbial, cancer related drugs, pesticides, and PXR mediators.

**Conclusions:**

Our results indicate that using microarrays and a supervised machine learning approach to predict chemical toxicants, their potential toxicity and mechanisms of action is practical and efficient. Choosing the right feature and classification algorithms for this multiple category classification and prediction is critical.

## Background

Efficient and precise evaluation of the potential hazards that drugs, environmental and industrial chemicals pose to humans and other organisms remains a challenge [1-4]. Traditional methods rely heavily on experimental animals and are extremely time consuming, inefficient and expensive. Only a fraction of commercially available chemicals have been tested because of the test difficulty of the traditional methods [5]. Therefore, it is essential to develop quick and efficient methods to test and predict the hazard and potential mechanisms of toxicity for chemicals.

Based on the concept that the similar gene expression patterns for similarly classified chemicals may indicate similar toxicity and underlying molecular mechanisms [6], scientists have used toxicogenomics strategies to predict toxicity of various compounds. For instance, gene expression profiles have been successfully applied to the classification of toxicants in rodents [7-11], and discriminate between hepatotoxic and nonhepatotoxic chemical compounds [12,13]. Similarly, this method was used to successfully distinguish genotoxic from nongenotoxic carcinogenetic chemicals by gene expression profiles in primary mouse hepatocyes [14]. Short-term transcriptional profiles have also been used to predict the long-term cancer-related safety of environmental and industrial chemicals [15-18].

Several strategies have been employed to classify expression profiles based on exposure to chemicals of known mechanisms of action [19,20]. Different feature selection and classification methods have been used for the classification and prediction of sample grouping based upon microarray data. Comparative studies of the algorithms have revealed that choosing appropriate algorithms for the classification of microarray data is important [21-23]. But there remains a a need to comprehensively compare feature selection and classification methods for the classification and prediction using toxicogenomics data. Another challenge of using microarray data for prediction is overfitting, where classification models may not correctly predict new data despite good performance on training datasets [23,24]. To overcome overfitting, it is essential to choose the right methods or develop new ones.

The liver is the major site of chemical metabolism and a principle organ affected by the toxicity of chemical compounds [12,25,26]. Primary cultured cells such as hepatocytes offer a convenient *in vitro* system that can easily be manipulated and used to screen chemicals for toxicity using different molecular and biochemical methods. Primary cell cultures can also reduce concerns regarding animal availability, cost, and welfare that affect in vivo studies [27]. There is a long history of using in vitro systems to screen for new drugs to treat human disease and to study cellular and molecular effects of different molecules [28,29]. In this study, we built a rapid system to classify chemical compounds based on gene expression profiles generated from *in vitro* cultured primary rat hepatocytes. Primary rat hepatocytes were exposed in triplicate to one of 105 compounds or controls for 24 h followed by microarray analysis of the chemical effects. A total of 105 compounds were divided into 14 classes based on their known functional properties, modes of action, and health and safety concern lists (Additional file 1: Tables S1 and Additional file 2: Table S2). The 14 classes included anti-microbial reagents, cancer-related drugs, energetics (explosives), halogenated contaminants, hormones and endocrine disruptors, inflammatory mediators, lipid mediators and peroxisomal mediators, metals, oxidative stress mediators, pesticides, ployaromatic hydrocarbons, pharmaceuticals and protective care products (PPCPs), and pregnane X receptor (PXR) mediators. Control samples were regarded as one class. Some categories had chemicals that shared similar structures and cellular effects (e.g., peroxisomal mediators), while other categories shared similar endpoints (e.g., cytotoxicity for cancer chemotherapeutic agents).

We examined whether we could use microarray technology to accurately classify and predict these 14 class compounds so that we can quickly predict the possible mechanisms and toxic effect of a new compound if its gene expression profile in rat hepatocytes is available. We extensively compared various normalization, feature selection and classification algorithms for the classification of the 105 chemicals into the 14 classes. The normalization methods included gene median value and control sample based normalization methods. Feature selection methods included principal component analysis (PCA), chisquare, gainratio, inforgain, relief, and SVM recursive feature election (SVM-RFE). Classification methods used included decision tree J48, random forest (RF), Naive Bayes (NB), simple logistic (SL), and two support vector machine methods, LibSVM and SMO. We also proposed a new feature selection algorithm called gradient method, which had a high training classification rate as well as prediction accuracy with the lowest overfitting rate. Biomarkers that can distinguish compounds into the 14 classes were identified that can be used to predict molecular and toxic actions of chemicals based on gene expression profiles.

## Results

### Effect of normalization methods on the classification accuracy of compounds into 14 classes

Microarray experiments were performed using Agilent rat whole genome array (4X44k) in order to identify biomarkers that would distinguish and predict which of 14 classes of compounds, including control classes, a chemical exposure belonged. Cultured primary hepatocytes were treated with 105 distinct compounds, as well as their respective vehicle controls, for 24 h after which total RNA was isolated for array hybridization (Additional file 1). At least three biological replicates for each compound were used and a total of 531 array samples were generated. The microarray data have been deposited in the GEO databases with assigned number GSE19662.

**Figure 1:**
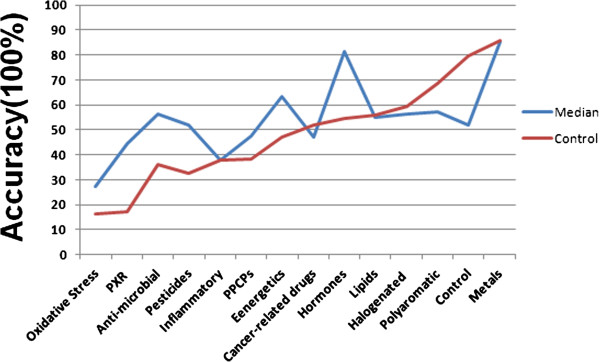
**Effect of normalization methods on the classification accuracy.** Microarray experiments were developed using Agilent rat whole genome array (4 X 44 k). Cultured primary hepatocytes were treated with distinctive 105 compounds (Additional file 1) as well as respective vehicle controls for 24 h; subsequently RNAs were isolated for array hybridization. 105 compounds treated samples and control samples were divided into 14 classes. Two normalization methods (median and control) based normalizations were compared for the classification accuracy of the 14 classes.

The experiments were conducted over two years. Dataset 1 is composed of a total of 168 array samples produced in 2007. Dataset 2 is composed of 363 array samples that were produced in 2008. For each dataset, representatives of each of the 14 classes were included. a complete set of 105 compounds were included. We first assessed the impact of normalization methods on the prediction accuracy of which class a chemicals belongs to out of the 14 classes. Two normalization methods were applied, median normalization and control normalization. For median normalization, the gene intensity was normalized by the median value of all the genes per chip and then a gene was normalized by the median value of the same gene across all the samples in the experiment. For control normalization, per chip normalization was conducted as in median normalization, then genes in treated samples and their matched control samples were normalized to the median values of the matched control samples. We compared the averaged prediction accuracies for each class, by averaging the prediction accuracies of 3 initial feature (gene number) filtering methods including One-Way ANOVA, Kruscal Wallis and One-way ANOVA unequal variance, four feature selection algorithms including ChiSquare, GainRatio, Inforgain, and Relief, and 5 classification methods including J48, RF, NB, SL and SMO. As depicted in Figure 1, the overall averaged prediction accuracies of the two normalization methods for all the classes were comparable. For an individual class, the median normalization method was generally better than the control normalization method with the exception of the control class whose prediction accuracy was higher using control normalization than median normalization.

### Effect of initial feature filtering methods on the classification accuracy

**Figure 2:**
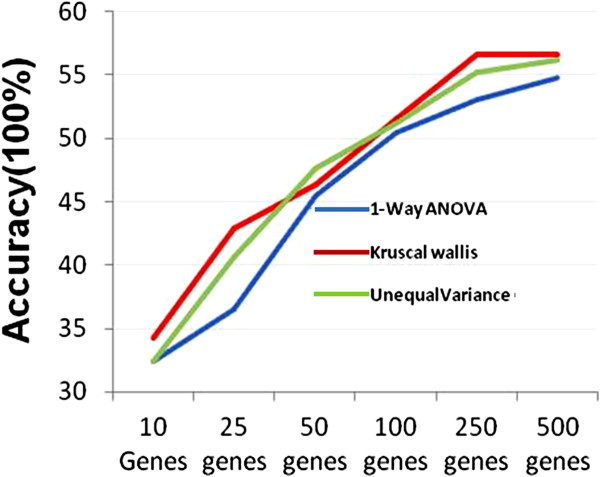
**Effect of initial feature filtering methods on the classification accuracy.** Three initial feature filtering methods including One-Way ANOVA, Kruscal Wallis and One-Way ANOVA unequal variance were compared for the classification accuracy for 14 class compounds. Different feature (gene) sizes to compare the mean prediction accuracies of 14 classes for each method, by averaging the prediction accuracy of different classification algorithms.

To find variable probe sets (or features) to separate microarrays into the 14 classes of compounds, we compared three initial feature filtering methods including One-Way ANOVA, Kruscal Wallis and One-Way ANOVA unequal variance. Using *p*-value ranked features, we chose different number of probe sets to compare the mean prediction accuracies for each method by averaging the prediction accuracy of the five classification algorithms decision Tree j48 (J48), RF, NB, SL and SMO. We observed that when the feature size was increased, the prediction accuracies for the three feature filtering methods increased as well (Figure 2). The averaged prediction accuracies of the three feature filtering methods were similar, but the Kruscal Wallis method performed slightly better than the other two methods.

### Effect of classification algorithms on the classification accuracy of 14 class compounds

**Figure 3:**
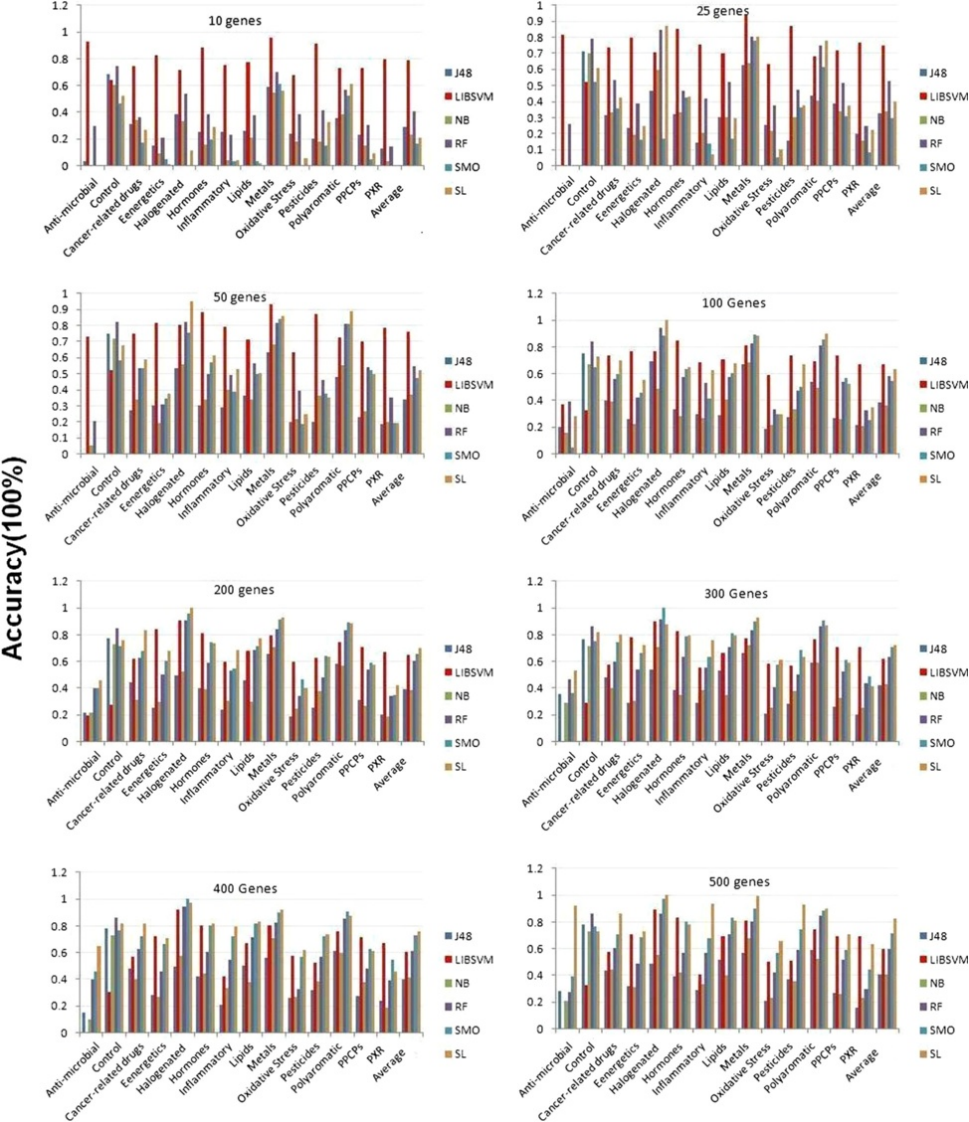
**Effect of classification algorithms on the classification accuracy.** Six classification algorithms including J48, LibSVM, NB, RF, SMO and SL were used for the comparison. The prediction accuracy shown here was the mean value by averaging the prediction accuracy of 6 feature selection methods including ChiSquare, GainRatio, Inforgain, PCA, SVM-RFE and Relief for different feature (gene) sizes (10, 25, 50, 100, 200, 300, 400 and 500).

Subsequently, we employed the median normalization and Kruscal Wallis feature filtering methods, we then focused on comparing a variety of classification algorithms. Six classification algorithms were used for the comparison. The prediction accuracy shown in Figure 3 was the mean value obtained by averaging the prediction accuracy of 6 feature selection methods including ChiSquare, GainRatio, Inforgain, PCA, SVM-RFE and Relief. We found that, in general, prediction accuracy for almost all class methods increased, with the exception of LibSVM, when the number of genes used increased. In contrast, LibSVM performed as well as, or better than, higher gene numbers when only a low number of genes were used (< 100 genes) (Figure 3). Regardless of gene numbers were used, J48 and NB always performed worse than the other methods. When the gene (feature) numbers were between 200 and 300, LibSVM, RF, SMO and SL methods performed similarly, with SL performing slightly better than the other three methods. When feature size reached ≥ 400, SL performed the best. By comparing the performances of these classification algorithms on individual classes, we observed a similar pattern as with the averaged prediction accuracy where LibSVM performed well when the feature sizes were small, yet SL and SMO performed well when more genes were used. Interestingly, for some compound classes such as PXR, LibSVM always performed the best regardless of feature size selected. However, LibSVM was the worst algorithm to predict the control class when the gene numbers were over 400 (Figure 3). Compound classes such as metals, halogenated contaminants, and polyaromatic hydrocarbons consistently exhibited higher prediction accuracy than other classes no matter what classification algorithms were applied. In contrast, certain classes such as anti-microbial and oxidative stress generally received lower prediction accuracy by all the classification algorithms.

### Effect of feature selection algorithms on classification accuracy

**Figure 4:**
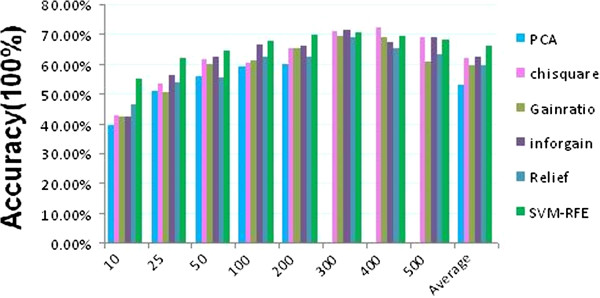
**Effect of feature algorithms on the classification accuracy.** The figure shows comparative prediction results for 6 feature selection methods, which include PCA, Chisquare, Gainratio, Inforgain, relief, and SVM-RFE. The prediction accuracy shown in the figure was mean values by averaging different classification algorithms including J48, LibSVM, NB, RF, SMO and SL for each feature size (10 to 500).

We compared prediction results for six feature selection methods: PCA, chisquare, gainratio, inforgain, relief, and SVM-RFE (Figure 4). The prediction accuracy was obtained by the averaging of values of six different classification algorithms: J48, LibSVM, NB, RF, SMO, and SL. Similar to previous observations (Figure 3), there was an increase in prediction accuracies when the number of genes increased. Overall, the SVM-RFE algorithm outperformed other methods no matter what gene number was used (Figure 4). The maximum gene number found for PCA was 200, but it was the poorest performing algorithm in comparison to other algorithms. Inforgain, Chisquare and Gainratio algorithms gave comparable performances while the relief algorithm generally performed slightly worse. However, when only 10 genes were selected as features, relief outperformed inforgain, chisquare and gainratio algorithms.

### The best models for the classification of 14 class compounds

**Figure 5:**
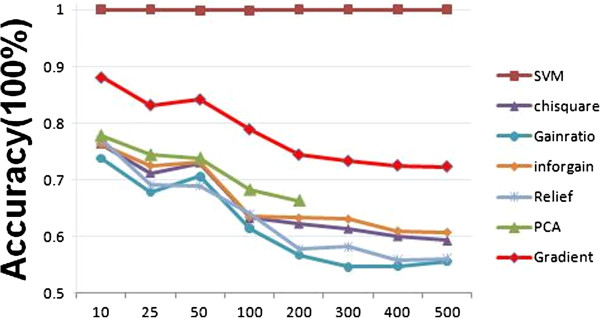
**The best models for the classification of 14 class compounds.** Seven feature selection methods, including PCA, Chisquare, Gradient, Gainratio, Inforgain, Relief, and SVM-RFE were used to compare their impact on the classifcation accuracy of 14 class compounds based on LibSVM classification algorithm. Different feature sizes (10 to 500) for each feature selection method were applied.

After comparing a series of methods, we found that using the SVM-RFE feature selection method and the LibSVM classification algorithm would achieve a high prediction accuracy for the 14 classes of compounds. Nearly 100% accuracy was observed for all feature sizes from 100 to 500 genes (Figure 5). In contrast, other feature selection methods such as chisquare, gainratio, inforgain, relief, and PCA had much lower accuracy when compared to SVM-RFE using the same LibSVM classification method. Overall, gainratio had the poorest prediction accuracy compared to other methods. Similar to what was observed in Figure 3, as more features were applied using LibSVM for classification, prediction accuracies for the 14 classes of compounds were reduced.

### Prediction of chemical classes using an independent dataset

To test the reliability of our classification models, we used the best model to train on dataset 2 to predict dataset 1. Dataset 1 was not used for training in the prediction of classes for dataset 2 because of the small size of dataset 2. When tested with SVM-RFE based on LibSVM, we obtained 100% training accuracy for dataset 2 using 200 gene features. However a low (65.1%) prediction accuracy was achieved suggesting that the method may be overfitting the data. To overcome these problems, we developed a new feature selection method called gradient method (see the Materials and Methods). We identified the best model for each feature selection method by comparing the different feature selection methods and classification algorithms in training on dataset 2. The best training accuracy by the feature selection methods was achieved using SVM-RFE (Table 1). The accuracy of relief, inforgain, chisquare, gainratio and gradient methods were comparable, but much lower than SVM-RFE (Table 1). The prediction accuracies of relief, inforgain, chisquare, gainratio were significantly lower than their respective training accuracies. The gradient method achieved the highest prediction accuracy, which was close to its training accuracy. When the gradient method was applied to all the samples (531) for training, the prediction accuracy was also comparable to other feature selection methods except SVM-RFE. When LibSVM classification was conducted, the training classification accuracy of gradient feature selection was much higher than other methods, with the exception of SVM-RFE (Figure 5).

**Table 1:** Prediction of 14 class compounds using independent dataset

**Feature selection methods**	**Feature number**	**Classification algorithm**	Training accuracy (%) (D2*)	Prediction accuracy (%) (D2 to D1*)
SVM-RFE	200	LibSVM	100	64.9
Relief	500	SL	83.7	72.6
Inforgain	500	SL	83.7	66.7
Chisquare	400	SL	82.6	72.6
Gainratio	500	SL	82.9	66.1
PCA	200	SMO	73.3	66.7
Gradient	300	SMO	85.7	79.7

*Dataset 1 (D1) has a total of 168 array samples what were produced in 2007, and dataset 2 (D2) includes 363 array samples that were hybridized in 2008. For each dataset, a complete set of 105 compounds were included.

**Figure 6:**
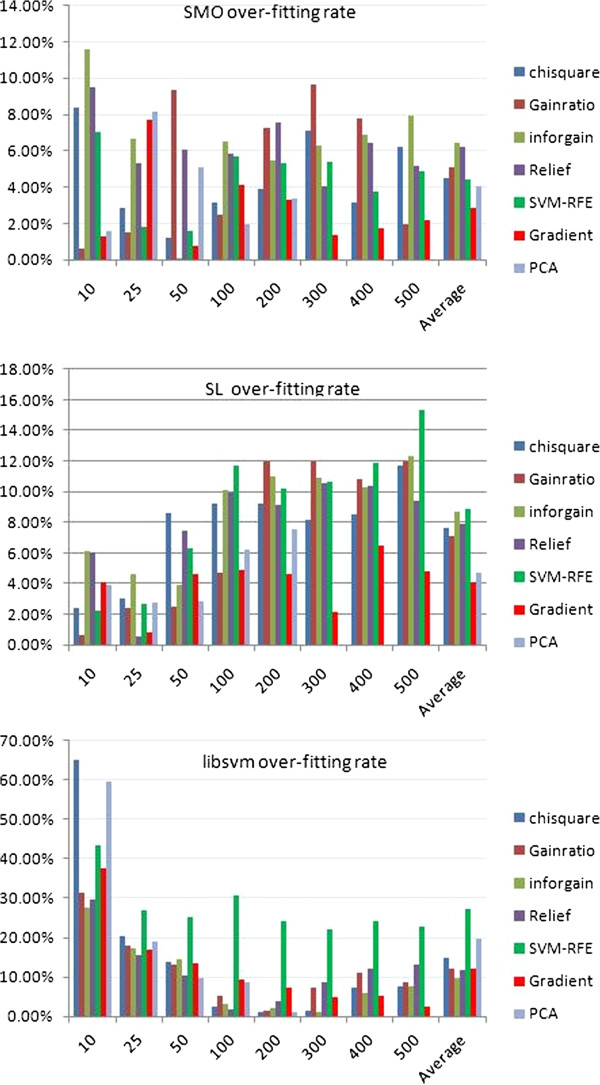
**Comparison of prediction overfitting rate of various feature selection methods.** The overfitting rates of different feature selection methods PCA, Chisquare, Gradient, Gainratio, Inforgain, Relief, and SVM-RFE over three classification algorithms, LibSVM, SMO and SL were compared. The overfitting rate was calculated by the percentage of the difference between the training accuracy and prediction accuracy of the summary of both the accuracies for a specific method.

Because of the difference between training and prediction accuracies, we further compared the overfitting rates of different feature selection methods over three classification algorithms (SMO, SL and libSVM) that could yield good classification accuracy. We found that the gradient feature selection method had the lowest overfitting rate for both SMO and SL classification algorithms (Figure 6). When LibSVM classification was applied, the gradient algorithm was also fairly low and was similarly low when the feature selection methods including Gainratio, Inforgain, and Relief were used. Inforgain had the highest overfitting rate for the SMO classification, followed by relief and gainratio feature selection methods. SVM-RFE had the highest overfitting rate when either SL or LibSVM classification was conducted. The algorithms had the second highest over fitting rates for SL and LibSVM were inforgain and PCA respectively (Figure 6).

### Gene expression pattern and functional analysis of gene markers

**Figure 7:**
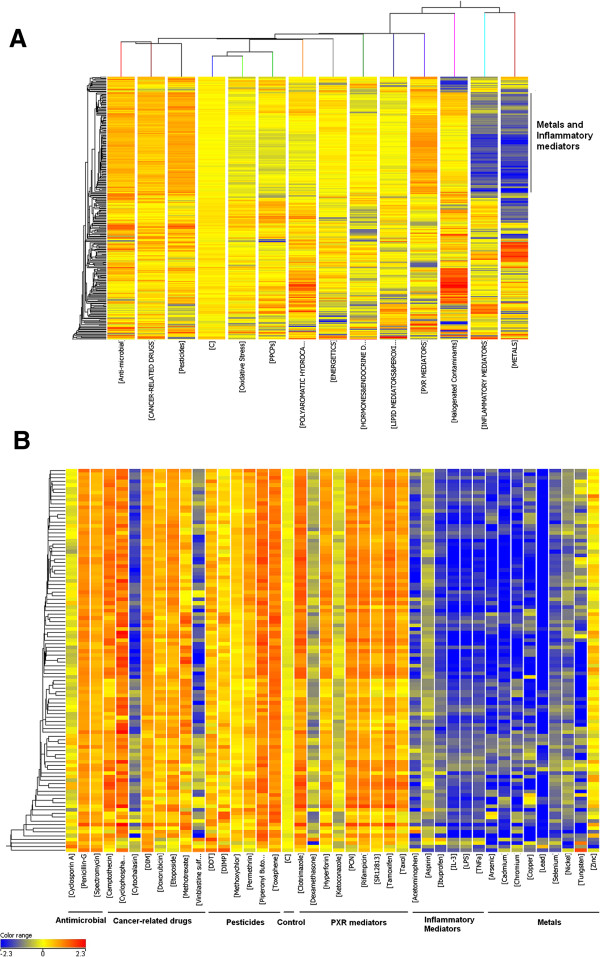
**Gene expression pattern analysis of biomarkers. A.** 300 transcripts (horizontal axis) resulted from the Gradient algorithm was used to perform a two-way hierarchical analysis across 14 classes (vertical axis). **B.** 104 transcripts (horizontal axis) were used to perform a hierarchical clustering across different compounds in the classes of antimicrobial, cancer related drugs, pesticides, PXR mediators, inflammatory mediators, and metals as well as control (vertical axis). An Euclidean distance algorithm was applied to calculate the distances between transcripts or between conditions. The relative level of gene expression is indicated by the color scale at the bottom of Figure 7B.

We chose 300 transcripts (gene features, Additional file 3) identified using the gradient algorithm to perform a two-way hierarchical cluster analysis across the 14 classes of compounds. Metal and inflammatory compound classes clustered together to form a separate group from other classes (Figure 7A). Antimicrobial, cancer-related drugs and pesticides clustered to form a distinctive subgroup (Figure 7A). Interestingly, 104 transcripts were strongly and specifically down-regulated by the metal and inflammatory compound classes, but were up-regulated by anti-microbials, cancer related drugs, pesticides, PXR mediators, and some halogenated contaminants.

Furthermore, we examined how each compound in the classes of metals and inflammatory mediators, antimicrobial, cancer related drugs, pesticides, and PXR mediators affected these 104 transcripts (Figure 7B). We observed that most of the compounds in the antimicrobial, cancer related drugs, pesticides, and PXR mediators classes up-regulated the expression of the transcripts, except cyclosprin A in the antimicrobial class, cytochalasin and vinblastine sulfate in the cancer-related drug class, dexamethasone and ketoconazole in the PXR mediators class. Most of the compounds in the metals and inflammatory mediators classes, in contrast, down-regulated the 104 transcripts with the exception of zinc.

Seventy-eight genes were well annotated in the 104 transcripts. Using Gene Ontology (data not shown) and the IPA function analysis tool, we found that cell cycle was the most significantly enriched functional term detected by both tools (Table 2). Surprisingly, over half the annotated genes belonged to the cell cycle process (42 genes). Other significant functional terms included cellular assembly and organization, DNA replication, recombination and repair, cellular movement, cell death, cellular compromise and cellular growth and proliferation. Most of the genes involved in the cell cycle process also play a role in one or more other functional processes listed in Table 2. Therefore, cell cycle was the most significant and crucial functional term in the 78 gene list.

**Table 2:** Functional analysis of biomarkers that distinguish 14 class compounds

**Category**	**p-value**	**No of genes**	**Molecules**
Cell Cycle	3.88E-29-2.23E-02	42	KIF23, KIF20A, CDC20, PTTG1, CCNB2, DSN1, KNTC1, MKI67, NUF2, AURKB, TTK, SKA1, BIRC5, RAD51, CCNA2, NEK2, TOP2A, CDKN3, KIF2C, ECT2, KIFC1, NCAPH, TRIP13, TACC3, ESPL1, CCNB1, RACGAP1, SPC25, PRC1, KIF4A, CKAP2, PLK1, FOXM1, BUB1B, CDC2, NCAPG2, BUB1, CCNE1, MCM2, KIF20B, NDC80, UBE2C
Cellular assembly and organization	8.61E-23-2.16E-02	33	KIF23, PTTG1, CCNB2, DSN1, AURKB, TTK, NUF2, SKA1, BIRC5, RAD51, CCNA2, NEK2, EZR, TOP2A, KIF2C, ECT2, NCAPH, KIFC1, TACC3, ESPL1, CCNB1, KIF4A, PRC1, SPC25, PLK1, CKAP2, BUB1B, NCAPG2, CDC2, BUB1, CCNE1, KIF20B, NDC80
DNA replication, recombination, and repair	8.61E-23-2.16E-02	44	KIF23, MCM6, CDC20, KIAA0101, PTTG1, CCNB2, DSN1, KNTC1, AURKB, TTK, NUF2, PBK, SKA1, BIRC5, RAD51, CCNA2, NEK2, TOP2A, KIF2C, ECT2, NCAPH, KIFC1, EXO1, MCM5, TRIP13, TACC3, ESPL1, CCNB1, KIF4A, SPC25, PRC1, CKAP2, PLK1, FOXM1, BUB1B, NCAPG2, CDC2, MCM3, BUB1, CCNE1, MCM2, POLA2, NDC80, TK1
Cellular movement	3.82E-18-1.73E-02	13	KIF23, KIF20A, CCNB1, CDC20, RACGAP1, KIF4A, PRC1, PLK1, AURKB, KIF20B, TOP2A, ECT2, KIFC1
Cell death	5.84E-08-2.44E-02	28	CDC20, PTTG1, TTK, NUF2, LMNB1, BIRC5, RAD51, CCNA2, NEK2, EZR, TOP2A, TACC3, CCNB1, ESPL1, PCSK9, SPC25, RRM2, PLK1, CKAP2, BUB1B, FOXM1, CDC2, NCAPG2, BUB1, CCNE1, MCM2, TK1, UBE2C
Cellular compromise	1.87E-05-1.73E-02	13	KIF23, TACC3, CCNB1, PTTG1, PLK1, CDC2, BIRC5, NEK2, EZR, TOP2A, NDC80, KIF2C, ECT2
Cellular growth and proliferation	5.43E-05-2.39E-02	32	KIF20A, KIF23, KIAA0101, PTTG1, MKI67, TTK, PBK, BIRC5, RAD51, CCNA2, NEK2, EZR, CDKN3, KIF2C, E2F8, MCM5, TACC3, ESPL1, CCNB1, PRC1, RRM2, PLK1, FOXM1, BUB1B, CDC2, MCM3, BUB1, CCNE1, MCM2, KIF20B, TCF19, UBE2C

Several gene families had multiple gene numbers participating in cell cycle and other functional processes (Table 2). Two such families were the kinesin family (20A (KIF20A), 20B (KIF20B), 23 (KIF23), 2C (KIF2C), and 4A (KIF4A)) and the cyclin family (cyclin A2 (CCNA2), cyclin B1(CCNB1), cyclin B2 (CCNB2) and cyclin E1(CCNE1)). The minichromosome maintenance complex component (MCM) family genes were also highly enriched (MCM2, MCM3, MCM5, and MCM6).

Pathway analyses also showed that cell cycle associated pathways were the most significant pathways in the gene list, which included mitotic roles of polo-like kinase, and cell cycle: G2/M DNA damage checkpoint regulation pathways (Table 3). Cancer-related pathways (pancreatic adenocarcinoma signaling, hereditary breast cancer signaling, and role of BRCA1 in DNA damage response) were also highly enriched in the gene list. Several genes were involved in multiple pathways listed in Table 3, and they were CCNB1, CCNB2, cell division cycle 2, G1 to S and G2 to M (CDC2) and RAD51 homolog (RAD51). Our results indicate that cell cycle is an important process and its opposite regulation can help to distinguish different compound classes.

**Table 3:** Pathway analysis of biomarkers that distinguish 14 class compounds

**Ingenuity canonical pathways**	**-log (p-value)**	**Ratio**	**Molecules**
Mitotic roles of polo-like kinase	11.6	1.45E-01	KIF23, CCNB1, ESPL1, CDC20, PTTG1, PRC1, CCNB2, PLK1, CDC2
Cell Cycle: G2/M DNA damage checkpoint regulation	6.17	1.16E-01	CCNB1, TOP2A, CCNB2, PLK1, CDC2
ATM signaling	4.16	7.55E-02	RAD51, CCNB1, CCNB2, CDC2
Nicotinate and nicotinamide metabolism	3.03	2.94E-02	NEK2, PLK1, TTK, CDC2
Inositol phosphate metabolism	2.57	2.27E-02	NEK2, PLK1, TTK, CDC2
Sonic hedgehog signaling	2.15	6.06E-02	CCNB1, CDC2
Pancreatic adenocarcinoma signaling	2	2.59E-02	RAD51, CCNE1, BIRC5
Hereditary breast cancer signaling	1.85	2.33E-02	RAD51, CCNB1, CDC2
Role of BRCA1 in DNA damage response	1.62	3.28E-02	RAD51, PLK1

## Discussion

In this study, we compared the efficacy of a variety of methods including different normalization, feature selection, classification algorithms for the classification of gene expression profiles of 105 chemicals into 14 classes of compounds. Few reports have studied the impact of normalization methods on the classification accuracy based on microarray data. Since microarray experiments were analyzed over different time periods in our study, a batch effect could significantly affect our analyses as has been observed in other studies [30-32]. Removal of batch effects through normalization can be used to integrate different microarray datasets and improve prediction [33]. Therefore, we compared two normalization methods including a method based on median value to all samples and methods based on median value to control samples to remove batch effects. As expected, the control sample based normalization improved the classification accuracy of the control class since the batch effect of the samples in the control class was removed. Nevertheless, the average classification accuracy is comparable for these two methods, and for other compound classes, the classification accuracy based on median normalization was even higher. Our results indicate that in our case with more than 10 class compounds, the control sample based normalization did not improve results relative to a simple median normalization.

For a quick evaluation, we chose 24 h for our microarray test. Our previous experiments did find that there were more genes were changed at 24 h than 48 h [25]. For an *in vitro* study, time is an issue, we do not want to be too long to increase the cost and also not too short and some effects may not occur. We have added the discussion and the reference in the discussion part. Note also that primary hepatocytes rapidly adapt to cell culture changing from the in vivo liver cells they once were. Perkins et al. 2006 [34] found that this adaptation effect appeared to dominate chemical effects at 48 hrs.

To examine if the microarray data could be used to predict what chemical class the rat hepatocytes were exposed to, we compared different classification algorithms for their performance in predicting which of the 14 classes a chemical exposure belongs. We found that decision tree analysis always performed the poorest, which was consistent with other comparative reports [21]. NB method did not perform well in this study, although has it performed well in two class datasets [21]. Overall two SVM algorithms, LibSVM and SMO, performed very well. SVMs have been shown to have the best performance in other comparative studies using both gene expression [21,23,35] and proteomics data [36]. Interestingly, we found that LibSVM outperformed other classification algorithms when the gene feature size was small, and thus far we have not found any studies to report similar results. This is the reason why more features had less performance in Figure 5 and it did not happen in other classification algorithms. SL classification algorithm also performed fairly well in comparison to other algorithms. Although a few studies have demonstrated that SL is an appropriate method for the classification of gene expression data [37,38], there has been no comparison of this method to other methods using gene expression data. Our results offer some useful information on applying this method to multiple class prediction.

We also conducted a comprehensive comparison of various feature selection methods on the classification of the 14 classes of compounds. For training purposes, we found that SVM-RFE usually outperformed other methods as has been confirmed elsewhere [21,39,40]. However, when an independent dataset, dataset 1, was used for a prediction, SVM-RFE gave a high overfitting rate. It had the highest overfitting rate when either SL or LibSVM classification was performed. Considering that a high training accuracy does not entail a high prediction accuracy, we developed a new feature selection algorithm called gradient method to find reasonable variable features across multiple classes. The gradient method had a similar training accuracy when compared to most of the feature selection methods (Table 1). It also achieved the highest prediction accuracy using the independent dataset (Table 1) with the lowest overfitting rate (Figure 6). Although efforts to reduce the prediction overfitting based on microarray data have been made [41,42], our method is a valuable addition for selecting features to produce a more reliable prediction.

**Figure 8:**
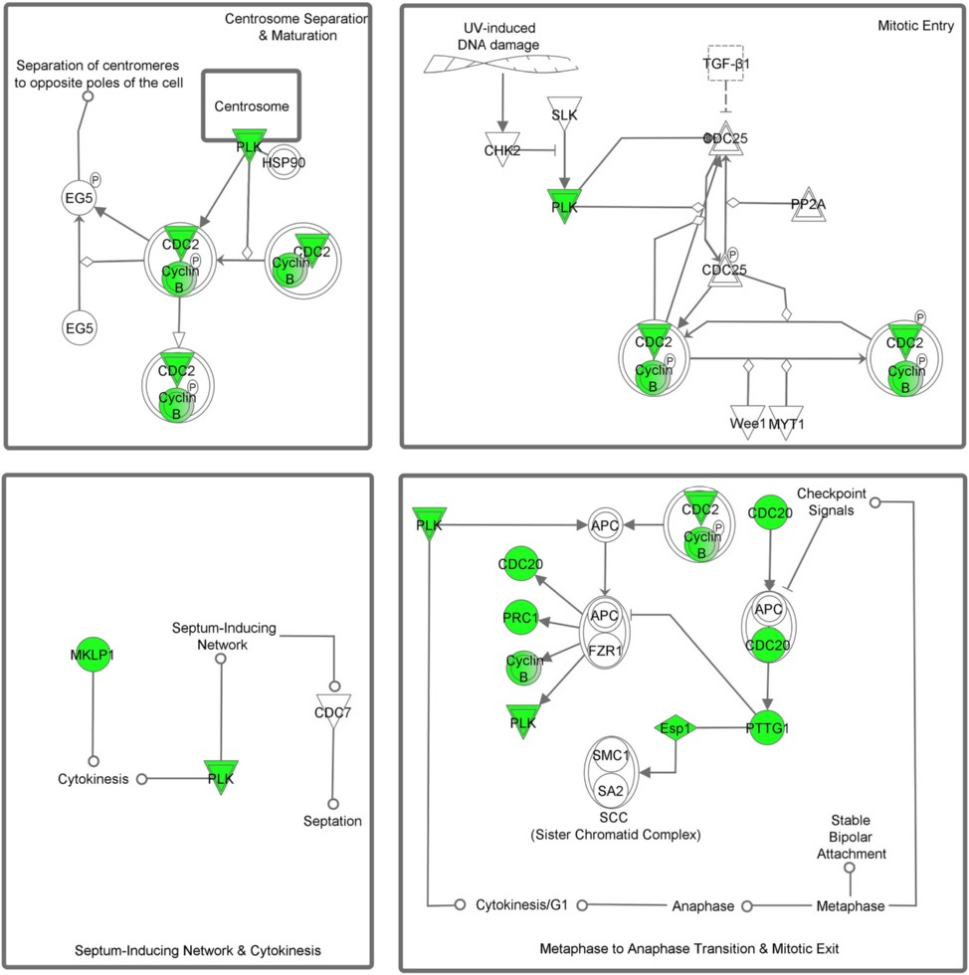
**Mitotic roles of Polo-like kinase pathway.** Most of the genes in the mitotic of Polo-like kinase pathway were down regulated (green color highlighted) by most of the compounds in the classes of metals and inflammatory mediators, but up regulated by most of the compounds in the classes of antimicrobial, cancer related drugs, pesticides, and PXR mediators.

**Figure 9:**
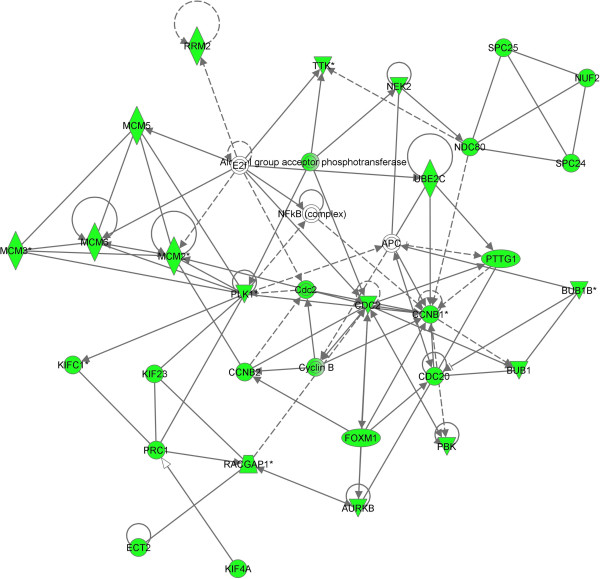
**Cell cycle related gene network.** A cell cycle network was constructed using Ingenuity knowledge base tool. Most of the genes in the network were down regulated (green color highlighted) by most of the compounds in the classes of metals and inflammatory mediators, but up regulated by most of the compounds in the classes of antimicrobial, cancer related drugs, pesticides, and PXR mediators. Nf-kB complex is connected with cell cycle genes.

Genes selected as biomarkers generally tend to have similar expression patterns relative to one or more classes of chemicals they are exposed to. When this common effect is seen between multiple classes a similar function may be impacted across the classes thereby providing some insight into mechanisms by which a chemical causes effects. We identified a cluster of 104 transcripts that were significantly down-regulated by metals and inflammatory compounds, but were up-regulated by anti-microbials, cancer-related drugs, pesticides, and PXR mediators. Over half of the genes in this cluster are involved in cell cycling. This result indicates that metals and inflammatory mediators may share similar activity. The inflammatory mediator class contained compounds such as aspirin, ibuprofen, IL-3, LPS, TNFα, have inflammation regulation function. A series of studies has also revealed that metal complexes could affect inflammation [43-45]. The reduction of cell cycle gene expression by both inflammatory mediators and metals suggests that these compounds may regulate inflammation by inhibiting the cell cycle process. An important pathway involved in the cell cycle is mitotic roles of polo-like kinase pathway was significantly impacted where more than half the genes in the pathway (PLK1, KIF23, CCNB1, ESPL1, CDC20, PTTG1, PRC1, CCNB2, and CDC2) were repressed by the compounds in the two classes (Figure 8). The polo-like kinases (PLKs) make up an evolutionarily conserved and newly emerging family of essential cell cycle regulators. PLKs regulate diverse cellular and biochemical events at various stages of the M-phase. They are required at several key points through mitosis, starting from control of the G2/M transition through phosphorylation of CDC25C and mitotic cyclins and in the DNA damage checkpoint adaptation to prevent entry into mitosis. At the beginning of mitosis, various proteins are recruited to the centrosome, a maturation process that requires PLK. PLKs are also required for the establishment of a bipolar spindle. They have a role in the metaphase to anaphase transition via its interaction with the APC/cyclosome [46] (Figure 8). Many of these cell cycle genes including MCM, cyclin and KIF family genes directly interact with each other and directly or indirectly interact with the NF-κB complex (Figure 9). NF-κB is a well known molecule that participates in the inflammation process and plays an important role in cell cycle [47,48]. The network analysis identified a possible new mechanism of the inflammation regulation through the cell cycle gene regulated NF-κB complex pathway [49]. Further studies are needed to explore how these cell cycle genes affect the NF-κB pathway.

We have manually divided the chemicals into 14 classes according to their known mode actions and toxicities. While imperfect, it is a simple and usable classification scheme. Based on the classification system and gene expression profiles, we have successfully developed a gene expression classifier to predict different compound classes. This gene list should have applicability in predicting what class a new compound belongs to when a gene expression profile available with the 300 genes.

## Conclusions

Our results indicate that using microarrays and a supervised machine learning approach to predict chemical toxicants, their potential toxicity and mechanisms of action is practical and efficient. Choosing the right feature and classification algorithms for this multiple category classification and prediction is critical.

## Methods

### Chemicals

Chemicals were purchased from Chem Service (West Chester, PA) and SRI International (Menlo Park, CA), Sigma-Aldrich (St. Louis, MO), and Fisher Scientific (Fair Lawn, NJ). The purity of all these compounds was equal or greater than 98%. Prior to testing, the compounds were made up and serially diluted in dimethyl sulfoxide, DMSO (Fisher Scientific, Fair Lawn, NJ). Metal compounds were prepared and serially diluted in 0.1 μm-filtered, ultra-pure water before testing. The 24-h toxicity of each compound was first determined in the human hepatocyte cell line HepG2 using the Neutral Red cytotoxicity kit (In Vitro Toxicology Kit Tox-4) obtained from Sigma-Aldrich.

### Isolation of hepatocytes

The primary rat hepatocytes, rtNHeps (AC-2630), isolated from male Sprague Dawley and its hepatocyte culture medium (HCM), and supplements and growth factors (CC-3198) were purchased from Cambrex BioScience (Walkersville, MD).

### Cell culture and Microarray experimental design

The primary rtNHeps cells were reconstituted in HCM supplemented with ascorbic acid, fatty acid-free bovine serum albumin, transferrin, insulin, recombinant human epidermal growth factor, hydrocortisone 21 hemisuccinate, Gentamicin sulfate, and Amphotericin B immediately upon receipt. An aliquot of the cell suspension was stained in a 0.05% Trypan Blue solution and counted under an inverted microscope. The cells were then seeded at 3 × 10^6^ cells per (Type 1 collagen-coated) T-75 flask. The flasks were left in a 37°C, 5% CO_2_ incubator overnight to allow for cell attachment.

The cells were replenished with fresh HCM and dosed in triplicate flasks with the non-toxic concentration of each compound (based on the experimentally determined 50% lethal concentration [LC50] value in HepG2 cells) at 1% DMSO (v/v) or with solution at 1% water (v/v). Another set of triplicate flasks were dosed with the appropriate solvent control. Hence, for every 3 chemicals and a solvent control, a total of 12 flasks were used. A total of 105 chemicals were used, and each chemical has 3 treatments plus controls (Additional file 4). Exposures lasted 24 h, after which RNA was isolated from the cells.

### Total RNA extraction

Total RNA was extracted from about 30 mg of cell pellet. Cells were homogenized in the lysis buffer with FAST Prep-24 from MP at speed 6.0/s twice, each for 30s before using RNeasy kits (Qiagen). Total RNA concentrations were measured using NanoDrop® ND-1000 Spectrophotometer (NanoDrop technologies, Wilmington, DE, USA). The integrity and quality of total RNA was checked on an Agilent 2100 Bioanalyzer (Palo Alto, CA). Nuclease-free water (Ambion) was used to elute total RNA.

### Microarray hybridization

Rat whole genome oligo arrays in the format of 4X44K were purchased from Agilent (Santa Clara, CA). Sample cRNA synthesis, labeling, hybridization and microarray processing were performed according to manufacturer’s protocol "One-Color Microarray-Based Gene Expression Analysis" (version 1.0). The Agilent One-Color Spike-Mix (part number 5188–5282) was diluted 5000-fold and 5 μL of the diluted spike-in mix was added to 1 ug each of the total RNA samples prior to labeling reactions. The labeling reactions were performed using the Agilent Low RNA Input Linear Amplification Kit in the presence of cyanine 3-CTP. The labeled cRNA from each labeling reaction was hybridized to individual arrays at 65°C for 17 hours using Agilent’s Gene Expression Hybridization Kit. After washing, the arrays were scanned at PMT levels 350 setting using GenePix 4200AL scanner (Molecular Device Inc.), the Feature extraction software (V. 9.5.1) from Agilent was used to automatically find and place microarray grids, reject outlier pixels, accurately determine feature intensities and ratios, flag outlier pixels, and calculate statistical confidences.

### Microarray data analysis

Microarray data analyses were processed with GeneSpring version 7.0 and 10.0 (Agilent). The sample quality control was based on the Pearson correlation of a sample with other samples in the whole experiment. If the average Pearson correlation for a given sample with other samples was less than 80%, the sample was excluded for further analysis. If the scanned intensity was less than 5.0 for a probe, it was transformed to 5. A per chip (within) array normalization was performed using 50 percentile values of all the probe values in the array. Per gene (between) array normalization was also applied using either the median value of a gene across all samples (median based normalization) or relative control samples (control based normalization) in the experiment. Probe features were first filtered using flags. A "present" or "absent" flag was defined using the Agilent *Feature Extraction 9.5.1* software. Only a probe that had present flags in at least 50% samples of all the arrays was kept for further analyses. Data were subsequently log (base 2) transformed for statistical analyses. Initial feature filtering was conducted by One-Way ANOVA, Kruscal Wallis and One-Way ANOVA unequal variance with a cut off *p*-value less than 0.05.

### Feature selection

Several feature selection algorithms were used in the project including SVM-RFE, PCA, chisquare, gainratio, inforgain, and relief. SVM-RFE is an algorithm for selecting a subset of features for a particular learning task. The basic algorithm is the following: (1) initialize the dataset to contain features, (2) train an SVM on the dataset, (3) Rank features according to c_i_ = (w_i_)^2^, (4) eliminate the lower-ranked 50% of the features, (5) return to step 2. At each RFE step 4, a number of features are discarded from the active variables of an SVM classification model. The features are eliminated according to a criterion related to their support for the discrimination function, and the SVM is re-trained at each step. All other methods were performed using Weka program [50].

### Classification algorithms

The classification algorithms used in the project included decision tree J48, random forest (RF), Naive Bayes (NB), simple logistic (SL), two support vector machine (SVM) methods LibSVM and SMO. In decision tree structures, leaves represent classifications and branches represent conjunctions of features that lead to classifications. Iterative Dichotomiser 3 (ID3) is an algorithm used to generate a decision tree [21] which is based on the Concept Learning System (CLS) algorithm. J48 is an improved version of ID3 algorithm. Several improvements are included in J48 algorithm, such as choosing an appropriate attribute selection measure, and handling training data with missing attribute values, attributes with differing costs, and continuous attributes. RF is a classifier that consists of many decision trees. It outputs the class that is the mode of the classes output by individual tree [51]. NB is a rule generator based on Bayes's rule of conditional probability. It uses all attributes and allows them to make contributions to the decision as though they were all equally important and independent of one another [21]. SVMs are a group of related supervised learning methods used for classification and regression. To efficiently find the solution of the quadratic programming (QP) program, the SMO algorithm is one of the fastest SVM training methods. Like other SVM training algorithms, SMO breaks the large QP problem into a series of smaller possible QP problems. Unlike other algorithms, SMO tackles these small QP problems analytically and avoids using a time-consuming numerical QP optimization as an inner loop. LibSVM is another kind of SVMs, and was developed by [52].

### Error estimation

After running classification algorithms, we tested the cross-validation error estimation using 10-fold cross-validation with 10 iterations. The classification and prediction accuracy was calculated by the correctly predicted cases for each class or all the samples [21].

### Gradient feature selection algorithm

This is a feature selection method of multiple classes—at least 3 classes. The goal of feature selection is to choose those features whose expression is different in every class. We consider power of sample size as another important factor to conduct feature selection. Here is the whole procedure of Gradient Feature Selection Method.

First, we do statistical testing across all classes, then filter out the features whose p-value is bigger than 0.05.

Second, observation by observation, we perform the following steps:

1. Calculate the observation’s median value across samples in every class.

2. Sort these median values from smallest to largest.

3. Calculate the differences between each two sorted neighbor classes.

4. Calculate power of sample size between each two sorted neighbor classes. Calculate standard deviation of step 3.

5. Calculate geometric mean of step 4.

Rank each observation equal to the multiplication product of the results from step 3 and step 4. Last, we use the highest ranked ones to be candidate eigenvectors.

To have a clear understanding for this idea, let’s see an example, a matrix whose structure is like that showing in the following table.

In the matrix, there are *m* genes and *n* classes, and there are *x*_1_ samples in class_1_, *x*_2_ samples in class_2_,… and *x*_n_ samples in class_n_. Every gene has a median value across each class. Here is our rule of naming, for gene *y*, its median across samples 1 through *x*_k_ in class *k*, then it will be named g*y*_median_C*k*, so g2_median_C2 means median value of gene 2 across samples in class 2.

For each gene, after sorting the median values from smallest to largest, the fold-change of this gene between each pair of neighbor classes forms a stair, which can be diagrammed as below.

Fold change between two sorted neighbor classes decides the height of stair, and the power of sample size of the two classes decides if the marker is reliable enough. Here P stands for power, P_i_ stands for the power of sample size between class_i_ and class_i+1;_ while fold change *i* stands for the fold change between class_i_ and class_i+1._ For the gene shown above, its rank R as a bio-marker is:

$$\begin{array}{ll} \;R= & \text{stdev}\left(\text{Fold}\;\text{change}\;1,\text{Fold}\;\text{change}\;2,\ldots \text{Fold}\;\text{change}\;n-1\right)\\ & *\text{geomean}\left(P1,P2,\ldots ,\text{Pn}\right) \end{array}$$

### Gene functional analysis, pathway analysis and network construction

Significantly regulated probes were employed for two-way hierarchical clustering of both genes and samples using GeneSpring 10.0. A euclidean distance with average linkage was applied for the clustering. Gene functional categories were classified according to gene ontology [53] as well as the Ingenuity Pathway Analysis (IPA) tool (Ingenuity Systems, Redwood City, CA). A gene functional term enrichment *p*-value less than 0.05 was considered significant. Pathway analysis was performed using the IPA canonical pathways analysis tool. Similar to functional term analysis, a pathway with an enrichment *p*-value less than 0.05 was considered to be a significantly regulated pathway. Gene networks were constructed based on the IPA knowledge base. A score was assigned to a network according to the fit of the original set of significant genes. This score reflects the negative logarithm of the *p*-value that indicates the likelihood of the focus genes in a network being found together due to random chance [54].

## Competing interests

The authors declare that they have no competing interests.

## Authors’ contributions

WX participated in writing the manuscript and data analysis and paper submission. JA processed microarray data, conducted statistical data analysis, and developed new algorithm and software. YD conceived and directed the study, analyzed the results, and drafted the manuscript. DRJ and CYA conducted the *in vitro* experiments. XG performed the microarray hybridization. CZ participated in designing the algorithms in the manuscript. EJP conceived the study, coordinated the whole project. All authors read and approved the final manuscript.

## Supplementary Material

Additional file 1: Table S1 Chemical classes and their contained compounds. Click here for file

Additional file 2: Table S2 Compounds and their existing mechanism and toxic effects. Click here for file

Additional file 3: Table S3 300 markers to distinguish 14 classes. Click here for file

Additional file 4: Table S4 In vitro experimental design. Click here for file
